# How to use the nominal group and Delphi techniques

**DOI:** 10.1007/s11096-016-0257-x

**Published:** 2016-02-05

**Authors:** Sara S. McMillan, Michelle King, Mary P. Tully

**Affiliations:** School of Pharmacy, Menzies Health Institute Queensland, Griffith University, Gold Coast Campus, Gold Coast, QLD Australia; Manchester Pharmacy School, University of Manchester, Oxford Road, Manchester, UK; Manchester Academic Health Sciences Centre, Oxford Road, Manchester, UK

**Keywords:** Consensus methods, Delphi technique, Nominal group technique

## Abstract

*Introduction* The Nominal Group Technique (NGT) and Delphi Technique are consensus methods used in research that is directed at problem-solving, idea-generation, or determining priorities. While consensus methods are commonly used in health services literature, few studies in pharmacy practice use these methods. This paper provides an overview of the NGT and Delphi technique, including the steps involved and the types of research questions best suited to each method, with examples from the pharmacy literature. *Methodology* The NGT entails face-to-face discussion in small groups, and provides a prompt result for researchers. The classic NGT involves four key stages: silent generation, round robin, clarification and voting (ranking). Variations have occurred in relation to generating ideas, and how ‘consensus’ is obtained from participants. The Delphi technique uses a multistage self-completed questionnaire with individual feedback, to determine consensus from a larger group of ‘experts.’ Questionnaires have been mailed, or more recently, e-mailed to participants. *When to use* The NGT has been used to explore consumer and stakeholder views, while the Delphi technique is commonly used to develop guidelines with health professionals. Method choice is influenced by various factors, including the research question, the perception of consensus required, and associated practicalities such as time and geography. *Limitations* The NGT requires participants to personally attend a meeting. This may prove difficult to organise and geography may limit attendance. The Delphi technique can take weeks or months to conclude, especially if multiple rounds are required, and may be complex for lay people to complete.

## Introduction

The Nominal Group Technique (NGT) and the Delphi Technique are commonly referred to as consensus methods [1]. They aim to achieve a general agreement or convergence of opinion around a particular topic. Consensus methods are used in research that is directed at problem-solving, idea-generation, or determining priorities [2]. How consensus is defined and operationalised will vary from study to study, depending on the research objectives [3].

Consensus techniques such as the NGT and Delphi Technique are superficially similar to focus groups, a commonly used method in pharmacy practice research. All methods involve interaction within a group of participants, yet they can provide different outcomes. Focus groups are useful for investigating an issue in-depth, including the identification of problems, questions or significant issues. Consensus methods, however, raise potential solutions or answers to a question, which can then be prioritised or agreed upon. A key strength of consensus methods is the balanced participation from group members, unlike a focus group, whereby the facilitator must control for, and minimise the risk of, a dominant participant influencing the discussion. The structured format of consensus methods avoids this issue.

The aim of this paper is to provide an overview of the NGT and Delphi technique, including the steps involved and the types of research questions best suited to each method, with examples from the pharmacy literature. Therefore, it provides a useful starting point for pharmacy practice researchers new to consensus methods. Initially it describes how to conduct the NGT and Delphi Technique and provides examples of their use within the pharmacy context. Then, it considers the choice of experts for the panels and which types of research questions are best suited to which method.

## Nominal group technique

The NGT is a highly structured face-to-face group interaction, which empowers participants by providing an opportunity to have their voices heard and opinions considered by other members [4]. It was designed by Delbecq and Van de Ven and comprises four key stages: silent generation, round robin, clarification and voting (ranking or rating) [2]. These stages are briefly explained below.

### How to run the nominal group technique

While groups of between two and fourteen participants have been used in nominal group research (Table 1), a maximum of seven has been recommended [5]. A nominal group generally involves one to two questions which are sent to participants in advance. At the beginning of the meeting, participants are given up to twenty minutes to silently reflect or record their individual ideas in response to a question, i.e. silent generation [6]. The facilitator then asks one participant at a time to state a single idea to the group in a ‘round robin’ fashion. Participants are able to think of new ideas during this process, but must wait their turn before they can share with the group. This stage takes as much time as needed until no new ideas are forthcoming. It is recommended that there be no discussion at this stage and ideas are merely recorded verbatim on, for example, a flipchart or white board [2].

**Table 1:** Examples of studies using the nominal group technique

Authors	Aim			Groups^a^		Experts							Prioritisation or ranking
	Develop criteria or guidelines	Generate ideas	Problem solve	n	Size range	Pharmacists	Support staff	Academics/Researchers	Policy makers	Public	Doctors	Other	n
Bissell et al. [7]	∙			1	8	∙		∙	∙				NA^b^
Bond and Watson [22]	∙			1	13	∙		∙	∙	∙	∙		1
Bradley et al. [11]		∙		4	3–8	∙	∙						1
Cameron and MacKeigan [25]		∙		2^c^	–^d^							∙	2
Gastelurrutia et al. [24]			∙	2	7	∙		∙	∙			∙	1
Hutchings et al. [12]		∙		6^c^	4–9	∙	∙	∙	∙	∙			3
McMillan et al. [16]		∙		21	2–14	∙	∙			∙			1
Tully and Cantrill [23]	∙			1	10	∙		∙				∙	1

^a^n = number of nominal groups; size range = range of participant numbers for all groups

^b^Each item was assigned a similar weighting

^c^There was also an additional mixed-group event or discussion involving participants, followed by re-ranking

^d^Size range per group was not stated (total number of participants was 17)

The third stage is clarification of the ideas, which also provides the opportunity for a grouping step, where similar ideas are grouped together with agreement from all participants. Participants may also exclude, include or alter ideas, as well as generate grouping themes [7]. All ideas should be discussed to ensure participant understanding [2], thus enabling them to make an informed decision when they come to voting on ideas. Facilitators should emphasise that participants do not have to agree with all ideas listed as, at the end of the clarification stage participants are able to ignore ideas by voting on personal preferences. The round robin [8] and clarification phase [9] can take up to 30 min each. Facilitators should not direct participants during the clarification process, which may make this stage particularly difficult.

Participants are then provided with a ranking sheet, where they are asked to select their top preferences from the generated ideas. The number of items chosen by participants depends on the topic, but the ranking of five ideas is common in the literature [2, 5, 10]. The facilitator should specify that a number should be allocated to each selected item, with larger numbers reflecting greater importance [2, 5]. For example, for five ideas, the most important idea is scored five points. Although there is no anonymity for participants during nominal group discussions, individual scoring on a ranking sheet is confidential. Finally, the scores for each idea are summed and presented to the group for discussion. The timing for this stage is likely to depend on a number of factors, including the complexity of the topic and how many items need to be prioritised (the more items to rank, the harder the process and more time consuming it can become). Dening et al. [10] noted that voting could take up to 10 min to complete.

Ultimately, the time to complete one nominal group is variable, and depends on group size, how many questions are asked, and the type of participants involved. For example, Bradley et al. [11] documented a 2-h time limit to conduct a NGT for one question, whereas Hutchings and colleagues allocated half a day to conduct a NGT for two questions, followed by another half-day for a forum event [12] (see “Variations on the nominal group technique”).

### Variations on the nominal group technique

The NGT is a highly adaptable method, and can be used in addition to [7] or to inform, other methods, e.g. a discrete choice experiment [13]. NGT variations may be influenced by the available research and participant time, or the level of clarification, consensus or generalisability required for the topic. Ultimately, researchers need to ensure that the NGT is working for each participant group; it may be that stages need to be adapted. For example, for indigenous or culturally and linguistically diverse populations, it may be the cultural norm to discuss ideas as a group. Thus, a more appropriate variation to the process for generating ideas has been to combine the round robin and clarification stages [5]. Other variations could be in direct response to participant ability. If it is too difficult for participants to group similar ideas at the clarification stage then this grouping step could be avoided altogether [5]. While this may make it harder for participants to vote, i.e. there is a longer list of ideas to consider, it may cause less frustration for participants.

**Fig. 1:**
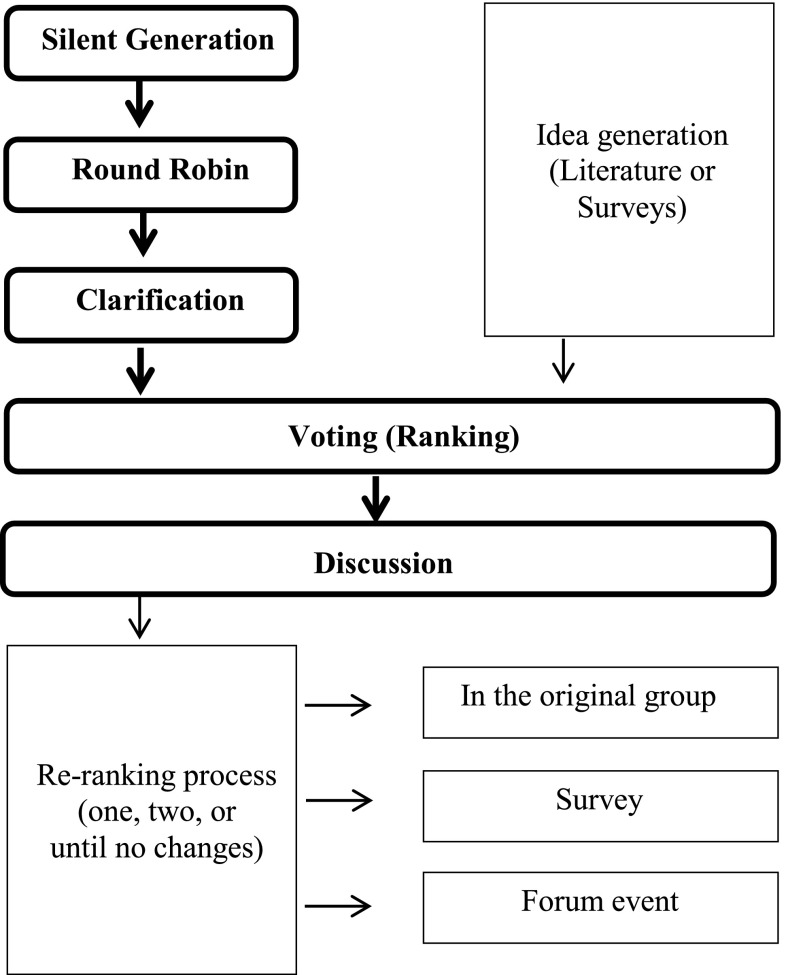
A simplified model of the NGT process and possible adaptions from the literature. *Traditional nominal group process is given in *bold*

Generally, variations are seen in relation to generating ideas, and how ‘consensus’ is obtained from participants, i.e. the ranking process (Fig. 1):

1. *Generating ideas* instead of silent generation followed by a round robin, ideas are obtained from a literature review[13], or exploratory surveys are used which could be viewed as a way to achieve greater consultation [14, 15];
2. *Ranking* this may be completed by either allocating a score[16] or by a rating on a Likert scale [15];
3. *Re*-*ranking* allowing participants to revise their original ranking, i.e. re-ranking, either in the original NGT meeting [9], via a secondary survey [14], or obtaining validation by sending a survey of nominal group results to other participants [15]. Alternatively, the re-ranking process could continue until no further changes are seen with the most important ideas [13].

Where separate nominal groups are held for similar participants, e.g. consumer groups, health professional groups or stakeholder groups, a mixed-forum event can provide the opportunity for consensus to be achieved by forming new groups with different participant types [12]. In a study that exemplified the use of a mixed forum event, Hutchings and colleagues asked previous participants to individually review the overall NGT results (overarching themes), and to rank the themes (pre-forum responses). At the forum, participants were asked to discuss the pre-forum responses in their newly allocated groups, which consisted of participants from differing backgrounds. Individuals were then asked to re-rank themes for a third time.

Other researchers have provided valuable information on important nominal group design considerations [3], its practical application [4] and method of analysis [5]. Black et al. [3] reviewed the literature to identify the evidence for certain ‘best practice recommendations’ for consensus methods. While that review provides some important considerations for researchers wishing to use these methods, the articles included are, at a minimum, over 15 years old. Using specific examples, Tully and Cantrill [4] discuss the steps involved in a nominal group, and guidance for researchers with respect to group composition. While a discussion of qualitative and quantitative analysis is also included, McMillan et al. [5] take this one step further in their paper by detailing the entire analysis process for researchers who undertake more than ten nominal groups.

### Applications to pharmacy research

The NGT has been applied in numerous healthcare settings, to develop guidelines [17] or explore opinions of different health professionals [18], lay people and carers [10, 19, 20], or to compare views of both parties [9, 21]. It is gradually building traction within the pharmacy setting, as seen in Table 1. Researchers have generated evidence based guidelines or criteria for pharmacy practice situations [7, 22, 23], informed practice change [11, 24] and the profession [12, 16] about particular topics, and identified attributes to be included when interviewing pharmacy students [25].

## The Delphi technique

Like the NGT, the Delphi Technique is a highly structured group interaction. However, the Delphi Technique uses interactions between group (called panel) members via questionnaires rather than face-to-face communication. This means that it preserves participant anonymity, if that is relevant. The Delphi Technique was developed by the Rand Corporation in 1953 [26] and uses a multistage self-completed questionnaire with individual feedback.

### How to run the Delphi technique

There is no standard method to calculate a panel size for the Delphi Technique; however, the aim of the study and available resources are important [27]. A sample of about fifteen has been suggested [26] but larger panels have also been used (Table 2). Inviting more participants increases the variety of expertise, but eventually leads to diminishing returns [3].

**Table 2:** Examples of studies using the Delphi Technique

Authors	Aim		No. of experts			Experts				No. of rounds	
	Generate ideas	Develop criteria or guidelines	Invited	Agreed	Completing	Pharmacists	Other healthcare professionals	Academics/Researchers	Other	Idea generation	Rating
Campbell et al. [31]		∙	305	305	79	∙	∙			0	2
Cantrill et al. [33]	∙	∙	–^a^	238	141	∙	∙			1	2
Cassar Flores et al. [36]		∙	18	18	18	∙	∙			0	2
Chan et al. [28]		∙	23	9	9	∙				0	2
Dean et al. [29]		∙	43	34	26	∙	∙			0	2
Mackellar et al. [30]		∙	38	–^a^	35	∙		∙		0	2
McBride et al. [39]	∙	∙	164	109	47	∙		∙	∙	1	2
McDermott et al. [37]	∙	∙	58	53	48	∙				1	2

^a^Number not given

The first-round questionnaire will present a series of statements that the respondent is asked to rate on a clearly defined Likert scale. The content of the statements may come from a variety of sources, singly or in combination, including the literature [28–30], clinical practice [31, 32], or from previous research findings, including NGT studies [30, 33]. Respondents are asked both to rate the item and to write free-text comments that, for example, explain their rating or express disagreement with the statement’s relevance. Reminders are sent to non-responders in the usual way.

**Fig. 2:**
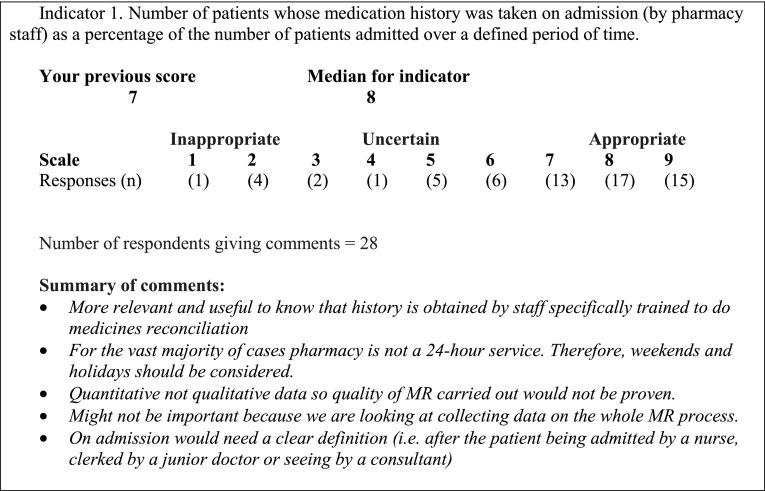
Example of individual feedback for a second-round questionnaire in a Delphi study [34]. Respondents were given a definition of appropriateness and asked to assess the appropriateness of indicators of medicines reconciliation

The responses to the first-round questionnaires are collated and used to create the second-round questionnaire. The latter presents the same statements as before, together with both the individual respondent’s rating and the median rating from the entire panel. A selection of the free-text responses is given, to represent the breadth of opinion. Respondents to the previous round thus get a personalised, unique questionnaire. Figure 2 provides an example of a statement from a second-round questionnaire seeking consensus on indicators for assessing medicines reconciliation processes [34]. After considering the group median and free-text comments, respondents re-rate the statements, by either giving the same rating as before or an amended rating. Respondents may give further comments about the statements if they wish.

The number of survey rounds is usually decided in advance and is dependent upon the level of dissension expected. In most studies, two rounds are used but occasionally, only a single round has been run [35]. More than two rounds increases panel attrition, so this is rarely done. The minimum time for a two-round Delphi can be as long as 30 days, although it may well take longer if multiple reminders are needed. The time required for the collation of responses and the creation of personalised second-round questionnaires should not be underestimated.

Often a 9-point Likert scale is used for the rating [29–31, 34], although 3-point [36], 5-point [28, 37] and 7-point [33] scales have also been used. The decision as to when consensus will have been reached must be made at the beginning of the study. For example, if the aim is to develop assessment criteria using the RAND 9-point scale [38], then consensus is reached that a statement is appropriate if the median score is greater or equal to 7, and it is inappropriate if the median score is less than or equal to 3. Disagreement is defined as where at least one third of respondents rate the statement at the opposite end of the scale to their peers. Such a finding would mean that consensus had not been reached.

### Variations on the Delphi technique

A common variant is for the initial study questionnaire to collect ideas in response to open questions [26]. Only subsequent questionnaires then ask respondents to conduct the rating process described above [33, 37, 39].

Examples of other modifications include researchers including only items that had failed to reach consensus in the second questionnaire (rather than all items, regardless of the ratings they received initially [29, 36]) and asking respondents to choose between alternatives (rather than rate items) for each question [32]. Traditionally, the questionnaire was sent by post, but more recently, e-mail has been used for the so-called e-Delphi Technique [40]. Emailed questionnaires per se are now so commonplace, that this is probably the new norm.

The RAND appropriateness method has been described as a variant of both the Delphi technique [38] and the NGT [3], as it has features of both. It involves participants reading a detailed literature review, followed by a traditional Delphi questionnaire. However, participants discuss the first-round results at a face-to-face meeting, followed by a second-round Delphi questionnaire and re-rating of the items.

### Applications to pharmacy research

An early use of the Delphi Technique in pharmacy practice research was in forecasting the future of hospital pharmacy in Australia [41]. It has been used to gain consensus on indicators for assessing prescribing appropriateness [33] or quality [31], criteria for safety features [36], clinically significant interactions [28] or aspects of student education [37] including communication skills [30] and professional engagement [35], or definitions, such as prescribing error [29]. This range of topics reflects the common use of the technique for the generation of clinical guidelines within the wider healthcare arena [3].

## Choice of experts

Experts, in the context of consensus methods, are those people who have knowledge about the topic of concern. Understandably, this is dependent upon the research aims and objectives, but such experts may not always be healthcare professionals. Given that greater importance has been placed on involving health consumers in research, consensus methods can be used to identify what is currently important to, or valued by, these experts. McMillan and colleagues, for example, explored the views of both the public and pharmacy staff on ideal community pharmacy services [16]. Therefore, their experts included people living with chronic conditions, their carers, and pharmacy staff who provided the relevant services. Campbell and colleagues, on the other hand, identified prescribing indicators that used data from dispensed prescriptions [31]. Therefore, their experts were the medical and pharmaceutical advisors who would be using the resultant indicators.

The NGT appears to be used more commonly with lay people than the Delphi Technique, although the reason why is not clear. Lay people may feel more comfortable participating in a face-to-face meeting, than in a relatively complex survey. For example, the NGT can be adapted to accommodate people with poor literacy [5]. The Delphi Technique has been used with patients in a small number of studies to prioritise outcome measures for clinical trials [42] and has begun to be used with members of the public (in this case, parents of children with attention deficit/hyperactivity disorder) in pharmacy practice research [43].

Power differentials between people in the NGT may mean that people with less power may feel unable to contribute their own views or contradict the views of someone more powerful. Therefore, it is usual for the experts in each meeting to be relatively homogeneous in status (see Table 1), such as running separate meetings for consumers and pharmacists [5]. This power differential may be less relevant for the Delphi Technique, as the experts are anonymous. Nonetheless, in those few Delphi studies that included both lay people and healthcare professionals, only patient data from the first-round questionnaire was sent to patients in the second-round questionnaire [44].

## Choice of consensus method

The decision whether to use the NGT or the Delphi Technique is influenced by various factors, including the research question, the perception of consensus required, and the associated practicalities and limitations such as time and geography.

If researchers are seeking to explore ideas in relation to a problem or question, this best aligns with the NGT, as idea generation is an integral part of this method. If researchers want to develop guidelines, a Delphi Technique involving experts who are likely to use the guidelines in question would be more suitable. The development of guidelines requires a more rigorous process, with consensus needed from a larger number of experts, which is easier with the Delphi Technique. This larger group may be needed to give authority to the final decision [3].

While some researchers have specified a numerical level of consensus when using the NGT, this is not well documented and would likely require further re-ranking beyond the initial steps. Alternatively, most researchers using the Delphi method will explicitly refer to a consensus value, i.e. a numerical level of agreement, determined by researchers in advance. Thus, it could be viewed that these two techniques sit along a spectrum of consensus, with a clearer description of the level of agreement thought to be given by the Delphi Technique [3].

As the NGT involves participants for only a few hours, results can be obtained quickly, suiting researchers who require a prompt result. It is particularly suited if participants are likely to only want to attend a single session compared to answering multiple questionnaires several weeks apart. The NGT requires face-to-face meetings, but this may be more culturally appropriate even if participants are at a distance. However, it may be more difficult to organise a nominal group meeting for a time that suits everyone. In contrast, the Delphi Technique is more flexible. The Delphi Technique, especially if conducted by email, is accessible to participants regardless of location, thereby avoiding travel expenses. Yet, this method can take weeks or months to conclude, especially if multiple rounds are undertaken.

## Conclusion

The NGT and Delphi Technique are both consensus methods that involve a group of ‘experts’ to generate ideas and determine priorities. The NGT has been used to explore consumer and stakeholder views, while the Delphi technique is commonly used to develop guidelines with health professionals. The NGT requires face-to-face discussion in small groups, and provides a prompt result for researchers. Alternatively, the Delphi technique uses questionnaires to preserve participant anonymity, can involve more participants but takes place over a longer time period.
